# Hypoxia-mediated apoptosis in oral carcinoma cells occurs *via *two independent pathways

**DOI:** 10.1186/1476-4598-3-38

**Published:** 2004-12-21

**Authors:** Nagathihalli S Nagaraj, Nadarajah Vigneswaran, Wolfgang Zacharias

**Affiliations:** 1 Department of Medicine, James Graham Brown Cancer Center, University of Louisville, Louisville, Kentucky 40202, USA; 2 Department of Pharmacology & Toxicology, University of Louisville, Louisville, Kentucky 40202, USA; 3 Department of Diagnostic Sciences, The University of Texas Health Science Center at Houston, Dental Branch, Houston, Texas 77030, USA

## Abstract

**Background:**

We are attempting to elucidate the mechanism of apoptotic cell death induced by hypoxia in oral cancer cells. Since hypoxia can render solid tumors more resistant to radiation and chemotherapy, understanding the pathways involved in hypoxia-induced apoptosis of oral cancer cells would be of significant therapeutic value.

**Results:**

Here we showed that oral cancer cells from primary tumor and lymph node metastasis undergo apoptosis after 24 to 48 h of hypoxia. During hypoxic growth, an increase in caspase-3 proteolytic activity was observed, accompanied by the cleavage of PARP (poly (ADP-ribose) polymerase) indicative of caspase activity. In addition, hypoxic stress also lead to activation of caspase-8, -9, and -10 but not -1, elicited the release of cytochrome C into the cytosol, and resulted in internucleosomal DNA fragmentation.

**Conclusion:**

These results show that hypoxia-induced apoptosis in oral carcinoma cell lines relies on both intrinsic (mitochondrial) and extrinsic (cell death receptor mediated) pathways. This novel evidence will assist in designing more efficient combination chemotherapy approaches as promising strategy for the treatment of oral cancers.

## Background

Oral cancer is one of the 10 most frequently occurring cancers worldwide, and its incidence in Europe and the United States ranges from 2% to 6% among all cancer patients [1,2]. The 5-year survival rate of less than 50% has not substantially improved over the past several decades, since many oral carcinomas respond poorly to chemotherapy approaches and their responses to radiation therapy have been highly variable.

Hypoxia, a reduction in the level of tissue oxygen tension, occurs during acute and chronic vascular disease, pulmonary disease and cancer, and can lead to apoptotic or necrotic cell death [3,4]. Fast growing tumors become hypoxic because newly developed blood vessels are inefficient and have poor blood flow. Although hypoxia is toxic to both cancer cells and normal cells, tumor cells can undergo genetic and adaptive changes in response to hypoxia that allow them to survive and proliferate [5]. Thus, hypoxic growth can result in a tumor with more aggressive growth characteristics and more malignant phenotype [3]. Although micro-environmental irregularities in solid tumors have been well documented, little is known about how different types of tumor cell phenotypes tolerate and respond to these conditions.

Apoptotic cell death is controlled by pro-apoptotic caspases, proteases that are synthesized as inactive precursors and activated by proteolytic processing [6]. The apoptotic cascade can be initiated *via *two major pathways, involving either the release of cytochrome C from the mitochondria (mitochondrial pathway) [7] or activation of death receptors in response to ligand binding (death receptor pathway) [8]. Upon triggering of either pathway, caspases, the final executioners of apoptosis, are activated, causing degradation of cellular proteins and leading to typical morphological changes such as chromatin condensation, nuclear shrinkage, and the formation of apoptotic bodies [9]. Both pathways are differentially involved in the cellular response to diverse apoptotic stimuli [10,11]. The majority of chemotherapeutic agents trigger the mitochondrial pathway, but the death receptors have also been reported to be involved in chemotherapy-induced apoptosis [12].

Death ligands such as TNF-α or CD95L recruit, *via *the adapter molecule FADD, cytoplasmic monomeric initiator caspase-8 to their surface receptors, resulting in dimerization and activation of caspase-8 [13,14]. Active caspase-8 cleaves and activates downstream effector caspases including caspase-3, -6 or -7, which degrade a broad range of cellular proteins and trigger the appearance of the apoptotic morphology [6,15]. On the other hand, mitochondria are important regulatory sites of the apoptotic process [16]. Defects in mitochondrial function result in release of cytochrome C, which can associate with Apaf-1 (apoptosis protease activating factor) and pro-caspase-9. The observation that chemical inhibition of caspase-9 blocks hypoxia-induced apoptosis points to a role of the complex in hypoxia-induced apoptosis [17,18]. This activation complex results in auto-processing of caspase-9 and further activation of downstream caspases, such as caspase-3 [19,20]. Activation of caspase-3 has been linked to the proteolytic cleavage of cellular substrates including poly-ADP-ribose-polymerase (PARP) [21], and is also necessary for the nuclear changes and chromatin condensation associated with apoptosis [22].

The low oxygen tension in hypoxic tumors is known to interfere with the efficacy of chemotherapy or radiotherapy. Also, hypoxia-induced apoptosis may impose a selection pressure favoring growth of more resistant tumor cells. However, the factors leading to hypoxia-induced apoptosis and their relative contribution to intrinsic and extrinsic apoptotic pathways are not well characterized. In the present study, we determined which factors in the mitochondria-dependent and -independent apoptosis pathways are activated in oral cancer cells. We observed that hypoxia-induced apoptotic cell death occurs through activation of caspase 8, but also cytochrome C release, caspase-9 activation, and results in caspase 3 processing, PARP cleavage, and DNA fragmentation. These results suggest that hypoxia-induced apoptosis in oral carcinomas cells relies on both intrinsic (mitochondrial) and also extrinsic (cell death receptor mediated) pathways.

## Results

### Hypoxia condition

Direct measurements of oxygen tensions in human tumors show a range of median oxygen tensions from 1.3 to 3.9 % (10–30 mm Hg), with readings recorded as low as 0.01 % (0.08 mm Hg) under severe hypoxia, whereas in normal tissues O_2 _levels can range from 3.1 to 8.7 % (24–66 mm Hg) [25]. Severe hypoxia stimulates cells to undergo apoptosis, whereas oxygen levels above 0.5 % prevent cell death, indicating tight regulation of cellular responses to the microenvironment. Critical O_2 _levels (hypoxic thresholds) characterize the upper limit of the hypoxic range below which activities and functions progressively become restricted. These levels can encompass O_2 _partial pressures from 35 mm Hg (start of reduced cell death in conventional photodynamic therapy or restricted efficacy of some immunotherapy) to 0.02 mm Hg; below this level, cytochromes *aa3 *and *c *are no longer fully oxidized [25]. During severe hypoxia or anoxia, a cascade of events is initiated that leads to global apoptotic cell death, thereby preventing the accumulation of cells with hypoxia-induced regulatory responses or mutations [26]. Thus, a condition of less severe hypoxia (1 %) was chosen to be able to monitor the response of viable cells under this condition.

### Morphological changes and DNA fragmentation during hypoxia

**Figure 1:**
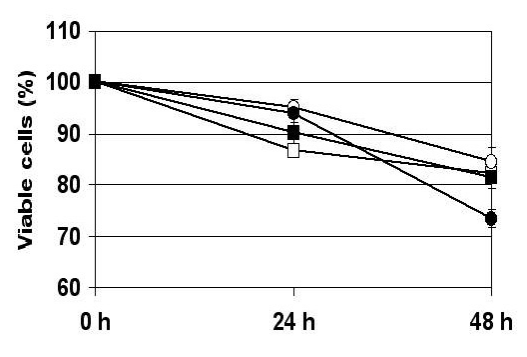
Viability assay for oral carcinoma cells under hypoxia. The numbers of viable (Trypan blue-excluding) cells were determined after 24 or 48 hours, and the percent of viability of hypoxic cells plotted relative to normoxic control cells; viabilities under normoxia were 100% for all cells. The results show the mean (± SD) of three independent experiments. 686Tu = open square; 686Ln = closed square; 1386Tu = open circle; 1386Ln = closed circle.

**Figure 2:**
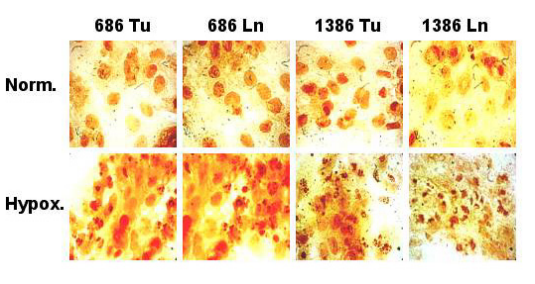
Hypoxia-induced nuclear TUNEL staining in oral carcinoma cells. The cells were incubated for 48 h in hypoxic or normoxic conditions, and photographs were taken after TUNEL staining of cells with DAB. Few apoptotic nuclei were observed in normoxic cells, but exposure to hypoxia for 48 h induced nuclear DNA condensation and fragmentation. Cells with nuclei showing strong chromatin condensation and nuclear fragmentation were considered apoptotic.

**Figure 3:**
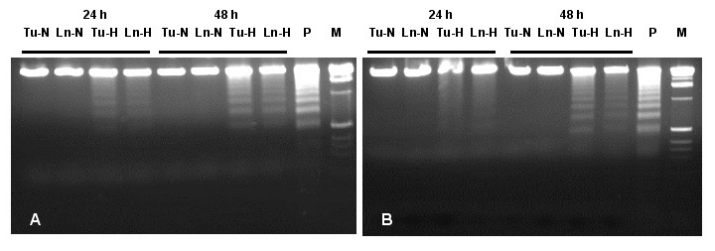
Effect of hypoxia on apoptosis induction as determined by DNA fragmentation. Nucleosomal DNA fragments in 686 (A) and 1386 (B) cells were analyzed by gel electrophoresis after 24 or 48 h hypoxia. Apoptosis was confirmed by the appearance of a ladder of oligonucleosomal DNA. M, molecular standard; P, positive DNA ladder control.

Trypan Blue dye exclusion was used to quantitate the number of viable cells after 24 and 48 h of hypoxia. TUNEL assays were used to visualize apoptotic cells, and DNA fragmentation into oligonucleosome-sized fragments, indicative of apoptotic cellular death, was monitored by gel electrophoresis [27]. The percentage of viable cells was steadily reduced in hypoxia-treated cells compared to normoxic control cells (Figure 1). Whereas approximately 5 – 15 % reduction of viability was seen at 24 h, all hypoxic cell lines declined further at 48 h to 85 – 75 % viability compared to normoxic growth. Hypoxic growth induced morphological alterations typical for apoptotic cell death, as determined by TUNEL assays (Figure 2). The hypoxia-treated cells showed extensive nuclear dye staining indicative for DNA breakage and cell death. This effect was detectable at 24 h (data not shown) and very apparent after 48 h of hypoxia, when most of the treated cells showed the typical morphology of apoptotic nuclear condensation. In contrast, nuclear staining was much weaker and less pronounced for normoxic cells up to 48 h incubation. Hypoxic conditions induced the chromatin alterations typical for apoptotic cell death, as demonstrated by fragmentation of chromosomal DNA into nucleosomal DNA ladders (Figure 3). Exposure to hypoxia for 24 or 48 h resulted in a time-dependent increase in DNA fragmentation in all four oral cancer cells, whereas no internucleosomal DNA fragmentation was observed in the normoxic control cultures (Figure 3). It is also apparent that the two metastatic lines 686Ln and 1386Ln had less extensive DNA fragmentation compared to their respective primary tumor lines 686Tu and 1386Tu.

### Processing of caspases, PARP cleavage and cytochrome C release

**Figure 4:**
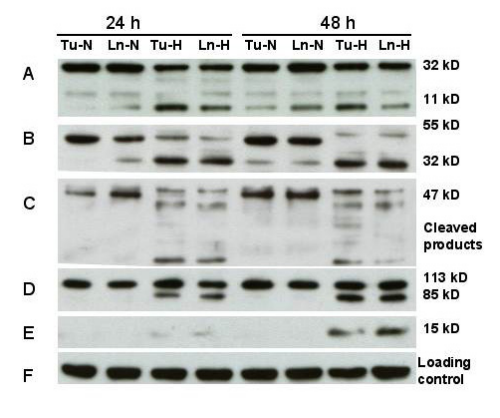
Response of apoptosis-related proteins to hypoxia. Western blot analysis of 686Tu (Tu) or 686Ln (Ln) cell extracts (30 μg each lane) after 24 or 48 hours of hypoxia (H) or normoxia (N) treatment. A: Cleavage of procaspase-3 (32 kD) into a 20 and 11 kD species; B: Cleavage of procaspase-8 (55 kD) into the 32 kD product (23 kD product not shown); C: Cleavage of procaspase-9 (47 kD) into lower mol. weight products; D: Processing of PARP (113 kD) into the typical 89 kD protein and a lower molecular weight product (not shown); E: cytochrome C (15 kD) release into cytosolic fraction; F: Re-probing for β-actin as an internal loading control. Data are representative of at least two independent experiments with similar results.

**Figure 5:**
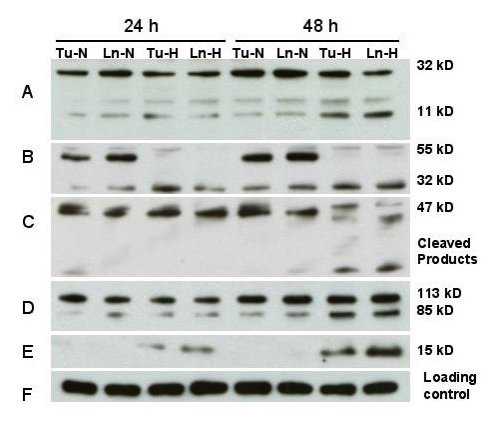
Response of apoptosis-related proteins to hypoxia. Western blot analysis of 1386Tu (Tu) or 1386Ln (Ln) cell extracts (30 μg each lane) after 24 or 48 hours of hypoxia (H) or normoxia (N) treatment. Other legend details are as for Figure 4.

The effects of hypoxia treatment on activation of key caspases and PARP in the four cell lines 686Tu/Ln and 1386Tu/Ln was determined by Western blotting using antibodies that recognize both full-length and cleaved proteins (Figures 4 and 5). Growth of cells under hypoxia caused a time-dependent processing of caspase-3, -8 and -9. For all four cell lines, hypoxia resulted in enhancement of procaspase-3 (32 kD) cleavage into the two immunoreactive fragments of ~20 and ~11 kD at the 24 and 48 h time points (Figures 4A and 5A). This treatment also resulted in cleavage of the 47 kD procaspase-9 to yield fragments of ~37 and ~20 kD, in parallel to caspase-3 cleavage (Figures 4C and 5C). Furthermore, caspase 8 was present primarily as ~55 kD pro-form in normoxic cells, whereas exposure to hypoxia resulted in its time-dependent processing to the ~32 kD active form (Figures 4B and 5B). These observations point towards involvement of both caspase-9 and caspase-8 in hypoxia-mediated cleavage of caspase-3 in all four cell lines. They suggest that a cascade of caspase activation occurs through the mitochondrial and also through the cell death receptor pathway in these cells in response to hypoxia.

To gain further insight into the role of mitochondria in this process, the extent of cytochrome C release under hypoxia was analyzed. Translocation of cytochrome C from the mitochondria to the cytosol was detected in all four cell lines after 24 h, and more pronounced after 48 h, of hypoxia (Figures 4E and 5E). This release of cytochrome C was a controlled event and not due to physical disruption of mitochondria, since no signal for intra-mitochondrial cytochrome oxidase could be detected in the same cytosolic fractions under these conditions (data not shown). Thus, these results demonstrate that hypoxia induced the release of cytochrome C from intact mitochondria.

Since we observed that hypoxia activated caspase-3 in the oral carcinoma cells, we investigated the cleavage of the caspase-3 substrate PARP under hypoxic versus normal growth. Clearly, cleavage of PARP, as indicated by a decrease in the full-length 113 kD protein and appearance of the 85 kD cleaved PARP product, was prominent in hypoxic cells, whereas it was almost completely absent in normoxia cells (Figures 4D and 5D). A small amount of cleaved PARP was already found after 24 h hypoxic conditions, and this effect was much more pronounced at 48 h. Only very small amounts of PARP cleavage product could be detected in the normoxic 1386 cell line pair, whereas for the 686 pair PARP cleavage appeared undetectable.

### Caspase activities during hypoxia-mediated apoptosis

As caspases are early effectors for triggering apoptosis, assays to determine caspase enzymatic activities further substantiated our findings that hypoxia-induced apoptosis occurs through both intrinsic (mitochondrial) and also extrinsic (cell death receptor mediated) pathways. We examined caspase proteolytic activities in cell extracts using fluorogenic peptide substrates specific for individual caspases. These substrates are conjugated with AFC or AMC and have aspartic acid residues at P1 positions, a requirement for caspase proteolysis. Cleavage of these substrates after the aspartic acid residue results in release of unbound AFC or AMC which can be monitored fluorometrically.

**Figure 6:**
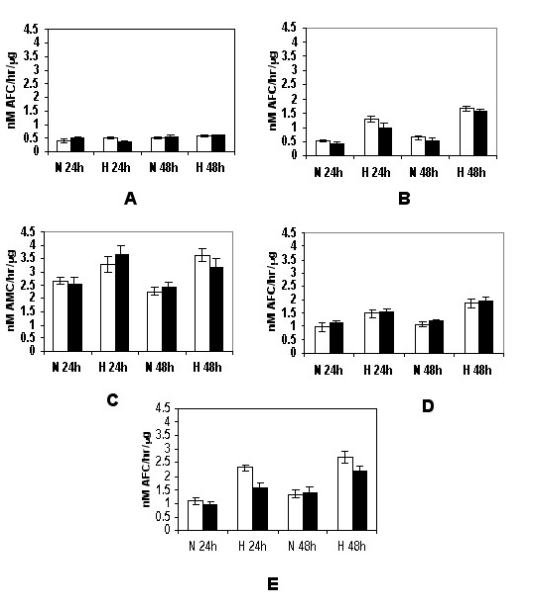
Hypoxia-stimulated caspase activities in 686 oral cancer cells. The 686Tu (open bars) and 686Ln (closed bars) cells were exposed to hypoxia or normoxic control growth for 24 or 48 hours, and induction of caspase activities were assayed as described in Materials & Methods. A: caspase-1; B: caspase-3; C: caspase-8; D: caspase-9; E: caspase-10. The means ± S. D. of three independent experiments are shown.

**Figure 7:**
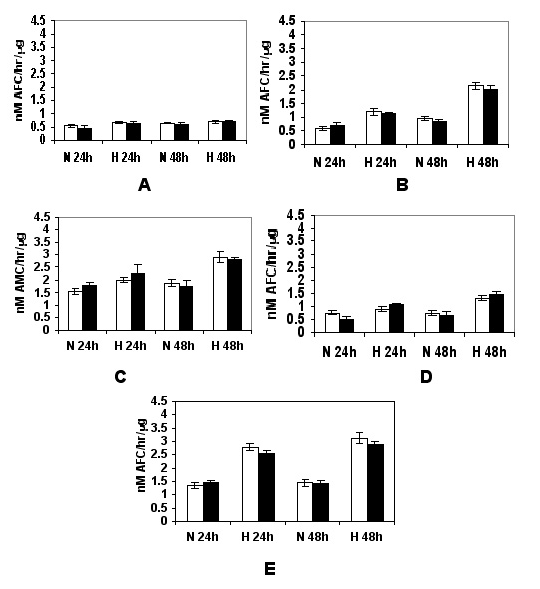
Hypoxia-stimulated caspase activation in 1386 oral cancer cells. The 1386Tu (open bars) and 1386Ln (closed bars) were analyzed; other legend details are as for Figure 6.

Detergent extracts prepared from cells after exposure to hypoxia or normoxia for either 24 or 48 h were tested for caspase cleavage activities, and specific inhibitors for control measurements were used as described in Materials and Methods. N-Ac-DEVD-AFC is cleaved by caspase-3 and -7, but may also be cleaved by other caspases, N-Ac-YVAD-AFC is cleaved by caspase-1, N-Ac-IETD-AMC by caspase-8, N-Ac-LEHD-AFC by caspase-9, and N-Ac-AEVD-AFC by caspase-10. Activities of caspase-3, -8, -9 and -10 were clearly and consistently elevated during hypoxia treatment for up to 48 h compared to normoxic growth for the 686 (Figure 6) and 1386 (Figure 7) cell line pairs. In contrast, only low caspase-1 activities were found for all four cell lines in normoxic condition, and these were not substantially altered after hypoxia challenge at any of the times examined (Figures 6A and 7A).

Overall, significant induction of hypoxia-mediated cleavage activities for the N-Ac-DEVD-AFC, N-Ac-IETD-AMC and N-Ac-LEHD-AFC substrates was detected in all four cell extracts, and induction of these activities correlated well with the levels observed for caspase-3, caspase-8 and caspase-9 protein expression and processing. Thus, induction of apoptosis was preceded by the activation of activator caspase-8, initiating receptor-mediated apoptosis, and caspase-9, initiating mitochondrial apoptosis, as well as the effector caspase-3.

### Effects of caspase inhibitors on caspase activity profile

**Figure 8:**
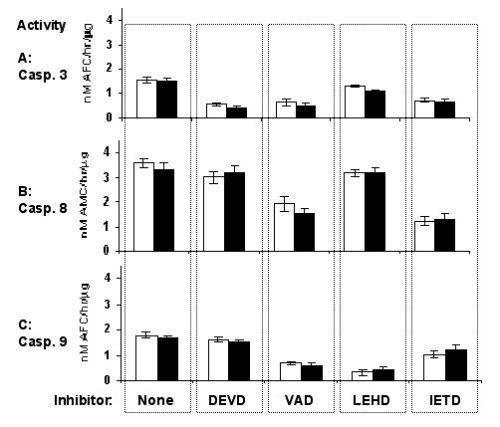
Prevention of hypoxia-stimulated caspase activities by intracellular caspase inhibitors. The 686Tu (open bars) and 686Ln (closed bars) cells were grown under hypoxia for 48 hours in the presence of different cell-permeable caspase inhibitors, and caspase activities were assayed as described in Materials & Methods. A: caspase-3 activity; B: caspase-8 activity; C: caspase-9 activity. The vertical dashed columns represent cell growth in the presence of the following caspase inhibitors: z-DEVD-fmk, caspase-3; z-VAD-fmk, pan-caspase; z-LEHD-fmk, caspase-9; z-IETD-fmk, caspase-8. The means ± S. D. of three independent experiments are shown.

We investigated the effects of individual cell-permeable caspase inhibitors on caspase-3, -8 and -9 activities during hypoxic growth for 48 hours (Figure 8). These inhibitors can enter viable cells and are covalently and irreversibly bound to their target caspases. Z-VAD-fmk was a pan-caspase inhibitor for all caspases analyzed; z-DEVD-fmk was inhibitor for caspase-3, z-LEHD-fmk for caspase-9, and z-IETD-fmk for caspase-8 activity. The presence of Z-DEVD-fmk clearly inhibited activity of its target protease caspase-3, but not caspases 8 or 9. The pan-caspase inhibitor z-VAD-fmk, as expected, diminished activities of all three tested caspases. Z-LEHD-fmk as inhibitor for caspase 9 did not affect activity of caspase-8, but did partially decrease caspase-3 activity. Finally, the z-IETD-fmk caspase-8 inhibitor also clearly decreased caspase-3 activity in addition to the target caspase. These data showed that both caspase-8 and caspase-9 contribute to the overall caspase-3 activity during hypoxic cell growth, and that those are the main caspases involved in hypoxia-mediated apoptosis activation pathways of these oral cancer cells. They suggest that both caspase-8 and caspase-9 activation pathways contributed to the activation of the major executioner caspases, such as caspases-3 and possibly caspase-7.

## Discussion

**Figure 9:**
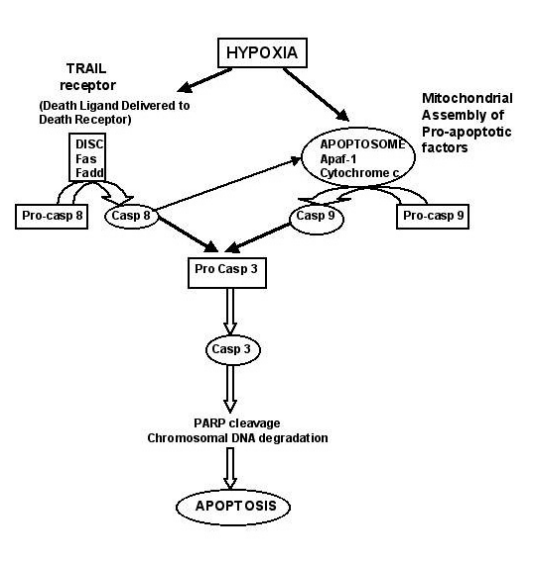
Potential pathways leading to apoptosis induction during hypoxia treatment. Hypoxia-induced apoptosis in oral carcinoma cell lines relies on both intrinsic (mitochondrial pathways) and also extrinsic (cell death receptor mediated) pathways. Key steps are activation of procaspase-8 or procaspase-9, then procaspase-3, and the subsequent cleavage of PARP by activated caspase-3, resulting in the induction of apoptosis.

The aim of this study was to identify factors which contribute to hypoxia-induced cell death in human oral cancer cells. The involvement of caspase pathways in induction of apoptosis of oral cancer cells during hypoxia was not previously determined. In the present study, we provide novel evidence for the participation of both initiator and effector caspases in this process (Figure 9). We showed that exposure to hypoxia elicits apoptotic cell death, and that this process relies on both intrinsic (mitochondrial) and also extrinsic (cell death receptor mediated) pathways. Our data showed that caspase-3, caspase-8, caspase-9, and caspase-10, but not the pro-inflammatory caspase-1, are activated during hypoxic growth. Activation of the executioner caspase-3 can be blocked in hypoxic cells by inhibitors of upstream caspases 8 or 9 during cell growth. We also observed that hypoxia-mediated apoptosis of oral cancer cells is associated with controlled cytochrome C release from mitochondria, proteolytic cleavage of PARP, and DNA fragmentation. Our results are in agreement with data on hypoxia-induced apoptosis in other cells [28-30]. In line with our data, others have observed activation of both caspase-9 and caspase-8 following hypoxic stress in animal models of brain ischemia [31,32]. Studies with caspase-9 knock-out mice demonstrated that caspase-9 is a critical upstream activator of the caspase cascade *in vivo *and may be essential for the processing of caspase-3 [33,34]. Also, earlier reports showed that chemical inhibition of caspase-9 protects against hypoxia-mediated effects [17,18].

On the other hand, it was suggested that key elements of the death receptor pathway are essential for hypoxia-induced apoptosis. The extrinsic pathway of apoptosis is initiated by death ligands, such as the Fas ligand or TRAIL (TNF-α related apoptosis inducing ligand), leading to the activation of caspase-8 and caspase-3 [35,36]. Recent studies indicate that DISC (Death Inducing Signaling Complex) formation precedes formation of Fas surface clusters, and that such clustering is dependent on DISC-generated active caspase-8 [37]. Also, TRAIL can induce receptor-mediated cell death selectively in tumor cells and is not active in non-malignant cells [38,39]. It was shown previously that in some tumor cells, only the receptor-independent mitochondrial pathway is activated during hypoxia without caspase-8 involvement [4]. On the other hand, there is recent evidence that TRAIL receptor-mediated apoptosis induction can be maintained and functional during hypoxic growth of tumor cells [39]. In view of the equal contribution of the caspase-8 and caspase-9 pathways established here, future work needs to examine the detailed mechanisms of receptor-mediated caspase activation with respect to TRAIL and death receptor-DISC-caspase-8 cascade, as well as the mitochondria-cytochrome C-caspase-9 cascade, and the possible involvement of HIF-1α as activator of caspases in OSCC cells. It was reported that hypoxia can induce upregulation of cell death receptors or death receptor ligands [40], and that inhibition of caspase-8 or FADD may interfere with hypoxia-induced apoptosis [18,41]. Our data also suggest that the activator caspase-8 is an integral component of the cell death-inducing mechanism in oral cancer cells, in agreement with other studies [32]. In receptor-mediated apoptosis, activation of caspase-8 represents a point of commitment to cell death. Thus, our data clearly show that in oral carcinoma cells two types of pathways are activated (Figure 9).

Hypoxia-induced caspase-3 activation and DNA fragmentation have been described by others recently [42,43], as well as caspase activation accompanying cytochrome C release from mitochondria [28-30]. Such findings correlate well with our studies showing that caspases-3, -8, and -9 activity and expression was significantly higher in hypoxic than in normoxic cells, and similar caspase activation was observed in the hypoxic cerebral cortex of newborn piglets [34]. In our cell system, PARP cleavage was observed within 24 h of hypoxia treatment and was accompanied by the appearance of a ~11 kD procaspase-3 cleavage product, suggesting activation of caspase-3. Caspase-3 is an executioner caspase that can be activated by a mitochondrial pathway involving release of cytochrome C [44]; alternatively, caspase-3 can also be activated by caspase-8 [45,46]. The results of the present study indicate that hypoxia-induced cleavage of procaspase-3 appears to be mediated by both caspase-9 and caspase-8 pathways.

Although cleavage of procaspase-9 was evident as early as 20 h into hypoxia treatment, it is possible that its activation is mediated by other caspases at earlier time points. Currently, possible involvement of other Bcl-2 family of apoptosis regulating proteins (e.g. Bad, Bag, Bak, Bik, etc.) in hypoxia-induced activation of the mitochondrial caspase cascade cannot be ruled out. The key regulator of hypoxia-induced cellular response is believed to be hypoxia inducible factor 1 (HIF-1). For all cell lines used here, we observed recently that there were several-fold increases in HIF-1α expression during hypoxia compared to normoxia (Wickramasinghe N, Banerjee K, Nagaraj N, Vigneswaran N and Zacharias W, manuscript submitted). HIF-1 can initiate apoptosis by inducing pro-apoptotic proteins such as BNIP3 or NIX, which will inhibit Bcl anti-apoptotic activity. It can also cause stabilization of wild-type p53 tumor suppressor, an effect that is lost in cells with pre-existing p53 mutations [26,47]. On the other hand, anti-apoptotic proteins, such as IAP-2, can be induced during hypoxia, whereas the pro-apoptotic protein Bax can be downregulated, leading to decreased accumulation of Bax in the mitochondria and thus decreased mitochondrial leakage and cytochrome C release [26,48].

It is apparent that during hypoxia, an intricate balance exists between factors that induce or counteract apoptosis, or even stimulate proliferation. More detailed studies are needed to define the precise mechanism for hypoxia-induced cleavage of procaspase-9 and procaspase-8; however, our results clearly demonstrate involvement of both caspase-8 and caspase-9 in hypoxia-mediated cleavage of caspase-3 and PARP. Because caspase-3 is a critical mediator of apoptosis [49] and correlates with the onset of apoptosis in oral cancer cells, it may be a potential marker for predicting response or resistance to chemotherapeutic agents in oral cancer.

The detailed temporal and spatial relationship of these events to other components of the apoptotic pathway including downstream caspases remain to be determined. Recently, the targeted elimination of oral squamous cell carcinoma cells by inducing apoptosis has emerged as a valued strategy to combat oral cancer [50]. Increased mitochondrial permeability is a crucial event in many types of chemotherapy-induced apoptosis and leads to release of cytochrome C from the mitochondrial intermembrane space. Our study confirmed that the release of cytochrome C was actually augmented during hypoxic growth, indicating a possible role of cytochrome C in hypoxia-mediated apoptosis. However, it also has been reported that certain anticancer drugs induce apoptosis in oral cancer cells but do not trigger cytochrome C release, thereby suggesting that cytochrome C can be an inducer-dependent phenomenon [51].

In some of the caspase cleavage assays, slightly lower activities were found for the metastatic Ln cells compared to the corresponding primary Tu cells. Also, the final appearance of nucleosomal DNA ladders is much more pronounced in both Tu cells than in their Ln counterparts. Although some of those differences were only minor, they are in line with previous studies which demonstrated much higher resistance of metastatic OSCC lines to TRAIL-induced cell death [38] and also to TNF-α-induced apoptosis [52] than their corresponding primary tumor lines. Such differential apoptosis sensitivity has also been observed recently in a different matched cell line pair form head & neck primary and metastatic carcinoma (UMSCC101A versus UMSCC101B; unpublished observations from this lab). On the other hand, a very apparent difference among the four cells is that caspase-8 activity is in general several-fold higher in the 686Tu/Ln pair than in the 1386Tu/Ln pair, which presumably is a reflection of the different pathologic histories of the two patients from which the respective tumor tissues were derived.

## Conclusions

In summary, we have reported that hypoxia directs apoptosis through mitochondria and cell death receptor mediated signaling pathways in oral cancer cells. We believe that this is the first report on caspase-dependent mechanisms during hypoxia in human oral cancer cells. Exposure to hypoxia lead to the activation of procaspase-9, -8, -3, and -10, cytochrome C release from mitochondria, with subsequent cleavage of PARP by activated caspase 3, finally resulting in the induction of apoptosis. The detailed molecular and sequential mechanisms of such hypoxia-induced caspase activation leading to apoptosis need further investigation. However, the knowledge of the relevant signaling cascades participating in this process can provide important insights in the mechanisms of acquired apoptotic deficiencies during malignant progression in poorly oxygenated oral carcinomas. It is well established that poor oxygenation of solid tumors is associated with poor prognosis. This may not only be due to direct effects of hypoxia on the efficacy on certain tumor treatment modalities, but also due to the evolvement of resistant tumor cells during the ontogenesis of a tumor under hypoxic conditions [39]. Our novel evidence, showing that hypoxia can induce apoptosis through both pathways, will assist in designing more efficient combination chemotherapy approaches as promising strategy for the treatment of oral cancers.

## Methods

### Cell lines

MDA-686Tu (686Tu) and MDA-686Ln (686Ln) cell lines were derived concurrently from the primary tumor and lymph node metastasis, respectively, of OSCC involving the left tonsillar fossa and posterior portion of the tongue in a 49 year old man (tumor stage T3N3B). MDA-1386Tu (1386Tu) and MDA-1386Ln (1386Ln) cell lines were obtained from the primary tumor and lymph node metastasis, respectively, of a 71 year old male patient with primary hypopharynx tumor (tumor stage T4N3B). All cell lines were generous gifts from Dr Peter Sacks, New York University, New York [23]. The cell lines were routinely maintained in DMEM/F12 50/50 mix (Cambrex BioScience, Walkersville, MD) containing 10% fetal bovine serum and 0.4 μg/ml hydrocortisone at 37°C with 5% CO_2_. All protocols for the use of human cell lines in this work were approved by the Institutional Review Boards of The University of Louisville and the University of Texas at Houston.

### Hypoxia exposure

Hypoxic conditions were produced by placing logarithmic phase subconfluent monolayer cultures, grown on 100 mm dishes, in a modular incubator chamber and equilibrating for 30 minutes with humidified gas containing 1 % oxygen, 5 % CO_2 _and 94 % nitrogen. The cell lines were maintained under hypoxic conditions for periods of 24 or 48 hours. Control cells were grown in normal oxygen conditions for the same duration. After incubation, media collection and cell harvesting were done immediately within 2–3 minutes to avoid adaptation of the cells to re-oxygenation.

### Cell viability assays

Determination of cell viability was done by Trypan Blue dye exclusion assay. Cells were grown in six-well plates (2 × 10^4 ^cells/well) in 3 ml medium to 70 % confluence, then washed and treated for hypoxia in DMEM/F12 medium with 10 % fetal bovine serum. For viability counting, cultures containing both dead and live cells from each well were collected, centrifuged, and resuspended in 0.5 ml FBS-free DMEM/F12. An aliquot of 0.1 ml was taken and incubated with 0.1 ml of Trypan Blue dye (0.4 %) for 5 min. Both live (unstained) and dead (blue) cells were counted in triplicate measurements from randomly selected fields in a hemocytometer.

### Protein extractions and Western blotting

Cultured cells were rinsed with PBS, gently scraped into 1 ml of PBS, and centrifuged at 4,000 rpm for 3 min. The pellets were resuspended into RIPA buffer (10 mM Tris-HCl, pH 7.4, 150 mM NaCl, 1 % Triton X-100, 0.1 % SDS, and 1 mM EDTA) containing fresh protease inhibitors (0.5 mM phenylmethylsulfonyl fluoride, 10 μg/ml aprotinin, and 2 μg/ml of both leupeptin and pepstatin) (all from Sigma, St. Louis, MO). Then, cell extracts were sonicated (Model 550 Sonic Dismembrator, Fisher Scientific, Pittsburgh, PA) for 1 min (1.0 sec on/0.5 sec off pulses) and cell debris was removed by centrifugation. Proteins were quantified using the Bradford protein assay kit (Bio-Rad, Hercules, CA) and compared with a γ-globulin standard curve. Equal amounts of total proteins were separated on a SDS-polyacrylamide gel and transferred onto a nitrocellulose membrane by electroblotting overnight at 20 V. Membranes were blocked in TBS-T (10 mM Tris-HCL, 150 mM NaCl, 0.25 % Tween 20, pH 7.5) with 5 % fat-free powdered milk at room temperature for 1 h. After rinsing membranes in TBS-T, the following primary antibodies were used: rabbit polyclonal IgGs for caspase-3 (H-277), poly(ADP-ribose) polymerase (PARP) (H-250), caspase-8 (H-134), caspase-9 (H-170) (all from Santa Cruz Biotechnology, St. Cruz, CA), or mouse monoclonal β-actin antibody (Sigma, St. Louis, MO). After incubation overnight at 4°C or 1 h at room temperature, the membranes were washed four times, 10 min each, in TBS-T. Secondary antibodies used were either horseradish peroxidase-conjugated goat anti-rabbit IgG or goat anti-mouse IgG (ICN, Costa Mesa, CA), followed by five washes with TBS-T. Bands were detected using the enhanced chemiluminescence ECL substrate (Amersham Biosciences, Piscataway, NJ). For β-actin detection, previously probed membranes were soaked in stripping buffer (70 mM Tris-HCl, pH 6.8, 2 % SDS, 0.1 % β-mercaptoethanol) at 60°C for 30 min and incubation as above.

### Caspase assays

After hypoxia treatment for 24 or 48-hours, treated and control cell cultures were rinsed once in cold PBS and collected in cold PBS by scraping. After centrifugation and removal of PBS, cell pellets were kept at -80°C until caspase assays were performed. The frozen pellets were resuspended in caspase lysis buffer (10 mM HEPES, pH 7.4, 2 mM EDTA, 0.1 % CHAPS) supplemented with protease inhibitors (5 mM dithiothreitol, 1 mM phenylmethylsulfonyl fluoride, 10 μg/ml pepstatin A, 10 μg/ml aprotinin, and 20 μg/ml leupeptin). Freeze-thaw cell lysis cycles were performed by alternatively transferring the samples from an ethanol/dry ice bath to a 37°C water bath five times. The supernatant was collected after 20 min of centrifugation at 12,000 rpm in a cold microcentrifuge. Assays were performed in caspase buffer (10 mM PIPES, pH 7.4, 2 mM EDTA, 0.1 % CHAPS, 5 mM dithiothreitol), to which 50 μM of substrate and 5 μl of protein extract were added to yield a final volume of 100 μl. Peptide substrates for caspase-3, N-acetyl-Asp-Glu-Val-Asp-AFC (DEVD-AFC), caspase-1, N-acetyl-Tyr-Val-Ala-Asp-AFC (YVAD-AFC), caspase-8, N-acetyl-Ile-Glu-Thr-Asp-AMC (IETD-AMC), caspase-9, N-acetyl-Leu-Glu-His-Asp-AFC (LEHD-AFC) (Biomol, Plymouth Meeting, PA) and caspase-10, N-acetyl-Ala-Glu-Val-Asp-AFC (AEVD-AFC) (Alexis, San Diego, CA) were dissolved in dimethyl sulfoxide. The respective specific inhibitors N-acetyl-Asp-Glu-Val-Asp-CHO (DEVD-CHO), N-acetyl-Tyr-Val-Ala-Asp-CHO (YVAD-CHO), N-acetyl-Ile-Glu-Thr-Asp-CHO (IETD-CHO), N-acetyl-Leu-Glu-His-Asp-CHO (LEHD-CHO) (Biomol), and N-acetyl-Ala-Glu-Val-Asp-CHO (AEVD-CHO) (Alexis, San Diego, CA) were used in control assay reactions. Assays were performed in black-wall, clear bottom plates using a Spectramax Gemini XS Microplate Spectrofluorometer (Molecular Devices); reading was at 500 nm after excitation at 405 nm for 7-amino-4-trifluoromethylcoumarin (AFC) and at 380 nm after excitation at 460 nm for 7-amino-4-methylcoumarin (AMC). The results were compared against AFC and AMC standard curves generated in parallel. Specific activity was expressed as units, with 1 unit defined as AFC or AMC release of 1 nMol/hour/μg protein.

### Cytochrome C release assays

Cells were collected at the indicated times and washed once in ice cold PBS. Cell pellets were resuspended in cytosol extraction buffer, and cytosolic extracts were prepared by the method described previously [24]. Western blotting for cytochrome C was done with mouse monoclonal anti-cytochrome C IgG (BD Biosciences-Pharmingen, San Diego, CA) as described above; the absence of intra-mitochondrial proteins was verified by blotting for mitochondrial cytochrome oxidase with mouse monoclonal anti-cytochrome oxidase IgG (BD Biosciences-Pharmingen, San Diego, CA).

### Hypoxic growth in presence of caspase inhibitors

Oral cancer cells 1386 and 686 were exposed to hypoxia for 48 hours in the presence or absence of individual cell-permeable inhibitors for caspase-3 (z-DEVD-fmk; 10 μM), caspase-8 (z-IETD-fmk; 20 μM), caspase-9 (z-LEHD-fmk; 20 μM), or pan-caspase (z-VAD-fmk; 10 μM) (all Santa Cruz Biotechnology, Santa Cruz, CA), and processed for caspase activity assays as above.

### Analysis of DNA fragmentation

Apoptotic cells were detected by *in situ *TdT-mediated dUTP nick end labeling (TUNEL) assays using the In Situ Cell Death Detection Kit POD, and nucleosomal DNA fragments detected with the Apoptotic DNA Ladder Kit (both from Roche, Indianapolis, IN). DNA fragments were resolved on 2 % agarose gels for visualizations of apoptosis-indicative DNA ladders.

## List of Abbreviations

AFC, 7-amino-4-trifluoromethylcoumarin; AMC, 7-amino 4-methyl coumarin; DAB, diaminobenzidine; DISC, death-inducing signaling complex; ECL, enhanced chemiluminescence; FADD, Fas-associated death domain protein; fmk, fluoromethylketone; PARP, poly (ADP-ribose) polymerase; TRAIL, TNF-α related apoptosis inducing ligand; TUNEL, TdT-mediated dUTP nick end labeling; z, benzyloxycarbonyl.

## Authors' contributions

NSN carried out the molecular and enzymatic studies and drafted the manuscript. NV participated in the design of the study, interpretation of collected data, and contributed to the manuscript preparation. WZ conceived and directed the study, contributed its design and coordination, and participated in the interpretation and final manuscript preparation. All authors read and approved the final manuscript.
